# The Novel Small Molecule STK899704 Promotes Senescence of the Human A549 NSCLC Cells by Inducing DNA Damage Responses and Cell Cycle Arrest

**DOI:** 10.3389/fphar.2018.00163

**Published:** 2018-04-16

**Authors:** Chan-Woo Park, Yesol Bak, Min-Je Kim, Ganipisetti Srinivasrao, Joonsung Hwang, Nak K. Sung, Bo Yeon Kim, Jae-Hyuk Yu, Jin Tae Hong, Do-Young Yoon

**Affiliations:** ^1^Department of Bioscience and Biotechnology, Konkuk University, Seoul, South Korea; ^2^Department of Biomedical Sciences, Protein Metabolism Medical Research Center, College of Medicine, Seoul National University, Seoul, South Korea; ^3^World Class Institute, Anticancer Agent Research Center, Korea Research Institute of Bioscience and Biotechnology, Daejeon, South Korea; ^4^Department of Bacteriology, Molecular and Environmental Toxicology Center, University of Wisconsin, Madison, WI, United States; ^5^College of Pharmacy and Medical Research Center, Chungbuk National University, Cheongju, South Korea

**Keywords:** lung carcinoma, STK899704, senescence, cancer therapy, cell cycle arrest, p53

## Abstract

The novel synthetic compound designated STK899704 (PubChem CID: 5455708) suppresses the proliferation of a broad range of cancer cell types. However, the details of its effect on lung cancer cells are unclear. We investigated the precise anticancer effect of STK899704 on senescence and growth arrest of A549 human non-small cell lung cancer (NSCLC) cells. STK899704 affected NSCLC cell cycle progression and decreased cell viability in a dose-dependent manner. Immunofluorescence staining revealed that STK899704 destabilized microtubules. Cell cycle analysis showed an increase in the population of NSCLC cells in the sub-G_1_ and G_2_/M phases, indicating that STK899704 might cause DNA damage via tubulin aggregation. Furthermore, we observed increased mitotic catastrophe in STK899704-treated cells. As STK899704 led to elevated levels of the p53 pathway-associated proteins, it would likely affect the core DNA damage response pathway. Moreover, STK899704 promoted senescence of NSCLC cells by inducing the p53-associated DNA damage response pathways. These findings suggest that the novel anti-proliferative small molecule STK899704 promotes cell death by inducing DNA damage response pathways and senescence after cell cycle arrest, being a potential drug for treating human lung cancers.

## Introduction

Cancers have many diverse characteristics, with many cellular pathways related with tumorigenesis and tumor maintenance (Bianco et al., 2006). Lung cancer is one of the most common cancer types worldwide. In 2016, lung cancer accounted for 14% of all new cancer cases. Approximately 85% patients with lung cancer have non-small cell lung cancer (NSCLC). According to the American Cancer Society, 27% cancer-related deaths in 2016 were caused by lung cancer (Siegel et al., 2016). Advances in our understanding of cancer biology have opened new avenues for the target-oriented anticancer drug development to control tumor growth. A recent study reported discovered ethyl(2-methyl-3-((E)-((naphtha(2,1-b)furan-2-ylca-rbonyl)hydrazono)methyl)-1H-indole-1-yl)acetate (STK899704; PubChem CID: 5455708) as a novel anti-proliferative agent that destabilizes microtubules, resulting in the mitotic arrest and apoptosis of a broad range of cancer cells (Sakchaisri et al., 2017). However, the details of the effect of STK899704 on lung cancer cells are unclear, providing us with a rationale for testing the anti-lung cancer effects of STK899704.

Microtubules are highly dynamic filamentous cytoskeletal fiber that play a major role in cellular processes including cell growth, division, and motility (Mukhtar et al., 2014). Microtubule polymerization can regulate its biological function (Waterman-Storer and Salmon, 1997). Therefore, disrupting the function of microtubules can be an important therapeutic strategy for tumor cells. Microtubule targeted antimitotic drugs are classified as microtubule stabilizing agents and destabilizing agents. Destabilizing agents bind one of the two domains of tubulin (vinca and colchicines), which leads to the abrogation of microtubule polymerization. Otherwise, most stabilizing agents bind to the taxoid binding site of beta tubulin, resulting in enhanced microtubule polymerization (Dumontet and Jordan, 2010). Both groups of microtubule targeted drugs arrest mitosis and induce cell death. STK899704 was reported as a microtubule depolymerizing agent possessing antimitotic effect in HeLa cervical cancer cells (Sakchaisri et al., 2017).

Development of anticancer drugs must consider a variety of possibilities. For example, apoptosis is most common anticancer drug target, but some cancers evade apoptosis by regulating apoptotic signaling pathways (Mohammad et al., 2015). For this reason, it is recommended to investigate non-apoptotic cell death pathways for cancer therapeutic targets, such as necrosis, mitotic catastrophe, autophagy, and senescence (Okada and Mak, 2004). Senescence is a permanent cellular stage induced by several processes, including cell cycle arrest (Campisi, 2007; Gorospe and Abdelmohsen, 2011). Cellular senescence can be determined by staining β-galactosidase in cells. The enzyme is a well-known and characteristic senescence indicator detectable at pH 6.0 that occurs only in senescence-induced cells (Debacq-Chainiaux et al., 2009). Cellular senescence is characterized by cell cycle arrest through the p53 and Rb pathways, enlargement of cell size, increase in the expression of senescence-associated secretory phenotype (SASP)-related proteins (interleukin [IL]-1β, IL-6, IL-8, CXCR2, etc.), and induction of mitotic catastrophe (Davalos et al., 2010; Gorospe and Abdelmohsen, 2011). Induction of cellular senescence arrests tumor progression and initiates caspase-independent cell death. Therefore, cellular senescence is a target for tumor therapy (Acosta and Gil, 2012). Several studies have reported that senescence-induced cell death exerts lower cytotoxic effects and induces lower immune stimulation than other cell death pathways (Canner et al., 2009; Kruse and Gu, 2009; Levine and Oren, 2009).

The p53 protein is a well-known senescence related and tumor suppressor protein that regulates essential processes for cellular integrity and maintenance (Kruse and Gu, 2009). p53 performs many functions that include maintenance of cellular homeostasis including expression of DNA repair-related proteins in the presence of DNA damage, induction of growth arrest by promoting cell cycle arrest, initiation of apoptosis; induction of antioxidant defense against reactive oxygen species (ROS), and induction of senescence and autophagy (Levine and Oren, 2009). Under normal conditions, p53 is regulated through ubiquitination by mouse double minute 2 homolog (MDM2), a p53 regulator protein (Canner et al., 2009). Several stresses induce p53 phosphorylation, including chemotherapeutic stress (El-Deiry, 2003). Active p53 regulates many pathways including activation of p21, which is a cyclin-dependent protein kinase (CDK) inhibitor acting on cyclin/CDK complex including cyclin A/CDK1 and 2 complexes (Kuo and Yang, 2008; Ruiz et al., 2008). Because p53 plays various pivotal roles, mutational inactivation and deletion of p53 lead to abnormal cellular outcomes including oncogenesis. p53 activity is not sufficient but is necessary for the senescence pathway (Ruiz et al., 2008).

In the present study, it is demonstrated that the novel small molecule STK899704 can effectively control human A549 NSCLC cells and its anti-tumor activity is induced by DNA damage responses and senescence, leading to death of NSCLC cells.

## Materials and Methods

### Xenograft

The experimental protocols were approved by the Institutional Animal Care and Use Committee (Permission No. KU 14141) and were performed according to the Konkuk University Animal Experimentation regulations. Male BALB/c nude mice (5 weeks old) were purchased from Nara Biotech Co., Ltd. (Seoul, South Korea). After 1 week of acclimation, the mice (*n* = 4 in each group) were inoculated subcutaneously in the back with a suspension (5.0 × 10^6^ cells/100 μl) of A549 cells as previously described (Kim et al., 2015). The length (*a*) and width (*b*) of the tumor masses were measured twice a week. The tumor volume was calculated using tumor volume = (a × b^2^)/2. When the tumor volume reached 50 mm^3^, the mice were divided into two groups consisting of four mice per group. Control group was orally injected with 100 μl of PBS. STK899704 group was orally injected with STK899704 at 1 μM once a week over 7 weeks, respectively.

### Cell Culture

The human NSCLC cell lines A549, NC1-H1299, and NCI-H460 were obtained from American Type Culture Collection (ATCC, Manassas, VA, United States). Lung cancer cells were cultured in RPMI medium (WelGENE Inc., Daegu, South Korea) supplemented with heat-inactivated 10% (v/v) fetal bovine serum (FBS; Hyclone Laboratories, Logan, UT, United States) and were incubated at 37°C in a humidified incubator containing 5% CO_2_. The cells were passaged no more than 20 times before using them in the study.

### Cell Viability Assays

Cell viability was quantified using a cell counting kit-8 (CCK-8) assay (Promega, Kumamoto, Japan). Approximately 0.7 × 10^4^ cells were seeded in each well of a 96-well plate containing 100 μL RPMI medium supplemented with 10% FBS, 100 μg/mL penicillin, and 0.25 μg/mL streptomycin and were cultured overnight. After 20 h of incubation, various concentrations of STK899704 were added to the wells and the cells were incubated further for the indicated time. Cell viability was analyzed by performing the CCK-8 assay according to the manufacturer’s instructions. Optical density was measured at 450 nm using a microplate reader (Apollo LB 9110; Berthold Technologies GmbH, Bad Wildbad, Germany). Also, cell viability was assessed by the MTS dye reduction assay, which measures mitochondrial respiratory function. Lung cancer cells were seeded (7 × 10^4^cells/mL) in 100 μL medium/well in 96-well plates, incubated overnight, and treated with various concentrations of STK899704, as described in the figure legends. Cell viability was calculated by assessing MTS metabolism as previously reported (Kwon et al., 2016). In brief, media samples (100 μL) were removed and incubated with 100 μL of MTS-PMS mix solution for 1 h at 37°C. Optical absorbance was measured at 492 nm using an ELISA reader (Apollo LB 9110, Berthold Technologies GmbH).

### Caspase Inhibitor Assays

The cell viability was analyzed with caspase inhibitors. A549 cells (0.7 × 10^4^ cells/ml) were seeded in each well of a 96-well plate and were treated with 100 nM pan-caspase inhibitors. After 2 h, the indicated concentration of STK899704 was added to each plate. Cell viability was analyzed by performing the CCK-8 assay after 24 h.

### *N*-Acetyl-L-Cysteine (NAC) Treatment

Cell viability was analyzed with NAC. A549 cells (0.7 × 10^4^ cells/ml) were seeded in each well of a 96-well plate and were treated with 10 mM NAC. After 1 h, the indicated concentration of STK899704 was added to each plate. Cell viability was analyzed by performing the CCK-8 assay after 24 h.

### Assessment of Cell Morphology

Cells were seeded in a 6-well plate at a density of 2.0 × 10^5^ cells/well and were incubated overnight. After 20 h of incubation, various concentrations of STK899704 were added to the wells and the cells were incubated further for 24 h. Cell morphology was determined using an inverted phase-contrast microscope (Eclipse Ts100; Nikon, Tokyo, Japan).

### Western Blotting

Cells were harvested and lysed. Cell lysates were clarified by centrifugation at 17,010 × *g* and 4°C for 30 min. Protein concentrations were estimated using the Bradford assay (Bio-Rad, Berkeley, CA, United States) with a UV/VIS spectrophotometer (Biowave; Biochrom, Cambridge, United Kingdom). Equal amounts of tissue lysates were resolved by performing sodium dodecyl sulfate-polyacrylamide gel electrophoresis on 10–15% gels. Protein bands obtained were transferred onto polyvinylidene difluoride membranes (Cat No. IPVH00010; Millipore, Billerica, MA, United States). The membranes were blocked with Tris-buffered saline containing Tween-20 [TBST; 2.7 M NaCl, 53.65 mM KCl, 1 M Tris–HCl (pH 7.4), and 0.1% Tween-20] and 5% non-fat dried milk for 1 h at room temperature. Next, the membranes were incubated overnight at 4°C with specific primary antibodies (diluted 1:2000 to 1:3000) to each target protein. After washing three times with TBST, the membranes were incubated with horseradish peroxidase-conjugated anti-rabbit or anti-mouse IgG secondary antibodies for 1 h at room temperature. After washing three times with TBST, the blots were exposed to WEST-ZOL (plus) Western Blot Detection System (iNtRON Biotechnology, Seongnam, South Korea). The following primary human monoclonal antibodies were used: poly (ADP-ribose) polymerase (PARP) 9542s, Caspase-9 9052s, Caspase-8 9764s, Caspase-3 9662s, Bax 2772s, Bid 2002s, Bcl-2 2876s, Bcl-xL 2762s, p53 9282s, pp53 2526s (all purchased from Cell Signaling Technology, Danvers, MA, United States), glyceraldehyde-3-phosphate dehydrogenase (GAPDH) sc-25778, cyclin D sc-25765, cyclin E sc-481, cyclin A sc-751, p21 sc-397, p27 sc-528 (all purchased from Santa Cruz Biotechnology, Santa Cruz, CA, United States), p62 p0067, and LC3B L8793 (both purchased from Sigma-Aldrich, St. Louis, MO, United States).

### Annexin–Propidium Iodide (PI) Staining

Cells (1 × 10^5^cells/well) were seeded in a 6-well plate and were incubated for 24 and 48 h. Next, the cells were treated with STK899704 in a dose-dependent manner and were incubated further for 24 h. The cells were then washed and harvested using trypsin-EDTA. Next, the cells were collected and were stained with Annexin V–fluorescein isothiocyanate (FITC) Apoptosis Detection Kit (BD Biosciences, San Jose, CA, United States) for 15 min. The proportion of apoptotic cells was determined flow cytometry analysis with an FACSCalibur device (BD Biosciences) with analysis using CellQuest software (BD Biosciences).

### Cell Cycle Analysis

Approximately 1 × 10^5^cells/well were seeded in 6-well plates and were incubated overnight. The cells were treated with different concentrations of STK899704 and were incubated further for 24 h. The cells were then washed, harvested, and fixed with ice-cold 70% ethanol at 20°C. After fixation, the cells were washed with phosphate buffered saline (PBS) and stained with PBS containing 50 μg/mL PI and 100 μg/mL RNase A for 30 min. The proportion of apoptotic cells was determined by performing flow cytometry analysis with the aforementioned FACSCalibur device and analyzed using CellQuest software.

### Reverse Transcription-PCR

Cells were lysed in 1 mL easy-BLUE Total RNA Extraction Kit (iNtRON Biotechnology), and RNA was isolated according to the manufacturer’s instructions. Oligo(dT)-primed RNA (5 μg) was reverse transcribed using M-MuLV reverse transcriptase (New England Biolabs, Ipswich, MA, United States). Reverse transcription polymerase chain reaction (RT-PCR) was performed in PCR Thermal Cycler Dice (Takara Bio Inc., Kusatsu, Japan) by using the following primer sets: *DR3* F 5′-CTA CTG CCA ACC ATG CCT AG-3′ and *DR3* R 5′-TCG CCA TGT TCA TAG AAG CC-3′, *TRAIL* F 5′-CAA CTC CGT CAG CTC GTT AG-3′ and *TRAIL* R 5′-GGT CCC AGT TAT GTG AGC TG-3′, *TRADD* F 5′-CTA TTG CTG AAC CCC TGT CC-3′ and *TRADD* R 5′-AGA ATC CCC AAT GAT GCA CC-3′, *FADD* F 5′-GGG GAA AGA TTG GAG AAG GC-3′ and *FADD* R 5′-CAG ATT CTC AGT GAC TCC CG-3′, *DR5* F 5′-CAC CTT GTA CAC GAT GCT GA-3′ and *DR5* R 5′-GCT CAA CAA GTG GTC CTC AA-3′, *DR6* F 5′-TGC AGT ATC CGG AAA AGC TC-3′ and *DR6* R 5′-TCT GGG TTG GAG TCA TGG AT-3′, *IL-1β* F 5′-GGA CCA GCT GAG GAA GAT GC-3′ and *IL-1β* R 5′-TCG TGC ACA TAA GCC TCG TT-3′, *IL-8* F 5′-GCT CTG TGT GAA GGT GCA GT 3′ and *IL-8* R 5′-GTT TTC CTT GGG GTC CAG AC-3′, *CXCR2* F 5′-CTC ATC TAC GCC TTC ATT GG-3′ and *CXCR2* R 5′GAA GTG TGC CCT GAA GAA GA-3′, and *E2F1* F 5′-GTG TGC ATG AGT CCA TGT GT-3′ and *E2F1* R 5′- -3′, *NOX1* F 5′-GCA AAT GCT GTC ACC GAT ATT C-3′ and *NOX1* R 5′-TGC AGA TTA CCG TCC TTA TTC C-3′, *NOX2* F 5′-GCT ATG AGG TGG TGA TGT TAG T-3′ and *NOX2* R 5′CTT CAG ATT GGT GGC GTT ATT G-3′, *NOX3* F 5′- TGA GGG TCT CTC CAC CAT ATT-3′ and *NOX3* R 5′ACT CCT CCT CTT CAT ACC AGT AG-3′, *SOD1* F 5′-TGG AAG TCG TTT GGC TTG T-3′ and *SOD1* R 5′-CAG CTA GCA GGA TAA CAG ATG AG-3′, *SOD2* F 5′GGG ATG CCT TTC TAG TCC TAT TC-3′ and *SOD2* R 5′-TAT AGA AAG CCG AGT GTT TCC C-3′, *TXN* F 5′-GAA GCT CTG TTT GGT GCT TTG-3′ and *TXN* R 5′-CTC GAT CTG CTT CAC CAT CTT-3′.

### Immunofluorescence Staining

A549 cells (1 × 10^6^cells/well) were seeded in a 6-well plate and were incubated overnight. Next, the cells were washed twice with PBS and treated with STK899704 for 24 h. After treatment, the cells were fixed and permeabilized using 4% formaldehyde for 15 min at room temperature. Non-specific sites were blocked by treatment with 1% bovine serum albumin (BSA) in PBS for 1 h at room temperature. Next, the cells were incubated overnight at 4°C with a mouse polyclonal primary antibody against γ-H2A.X (phosphor-S139, Cell Signaling Technology, 2577s), α-tubulin (Santa Cruz Biotechnology, sc-5286) diluted 1:50 in PBS containing 0.1% BSA, and were washed three times with PBS for 5 min per wash. Normal mouse IgG antibody was used a control. Next, the cells were incubated with FITC-labeled goat anti-mouse IgG secondary antibody (Merck Millipore, Darmstadt, Germany) diluted by 1:200 in PBS containing 0.1% BSA for 1 h at room temperature. After washing twice with PBS, the cells were stained with 4′,6-diamidino-2-phenylindole (DAPI; Sigma-Aldrich) for 10 s at room temperature. Fluorescence images were obtained using a BX61-32FDIC upright fluorescence microscope (Olympus, Tokyo, Japan) equipped with a 60× objective lens (green fluorescence, FITC; blue fluorescence, DAPI).

### SA-β-Galactosidase Staining

A549 cells (2 × 10^4^ cells/well) were seeded in a cell culture slide (30104; SPL, Seoul, South Korea) and were incubated overnight. After STK899704 treatment for 24 h, the cells were treated using Senescence β-Galactosidase Staining Kit (#9860; Cell Signaling Technology) to detect senescent cells according to the manufacturer’s instructions. The cells were incubated at 37°C in the absence of CO_2_ and light for at least 24 h. Stained cells were observed using a CX21 light microscope (Olympus). Cells in three random locations were counted.

### Analysis Autophagy-Related Factors by Chloroquine Diphosphate Salt (CQ)

The autophagy-related factors were analyzed in A549 cells treated with STK899704. A549 cells were seeded in each well of 6-well plates and treated with indicated concentration of STK899704 for 24 h. The expression levels of autophagy-related factors such as LC3B and p62 were analyzed by Western blot (Supplementary Figure S7). The cell viability was also analyzed in A549 cells treated with STK899704 and an autophagy inhibitor (chloroquine diphosphate salt; CQ). A549 cells (0.7 × 10^4^ cells/ml) were seeded in each well of a 96-well plate with 20 μM CQ. After 1 h, the indicated concentration of STK899704 was added to each plate. Cell viability was analyzed by performing the CCK-8 assay after 24 h (Supplementary Figure S7).

### siRNA Transfection

siRNA oligonucleotide against *p53* and scrambled siRNA were purchased from ST Pharm Co., Ltd. (Seoul, South Korea). The sequences of the siRNAs were: *p53* siRNA sense, 5′-CAC UAC AAC UAC AUG UGU A-3′; p53 siRNA antisense, 5′-UAC ACA UGU AGU UGU AGU G(dTdT)-3′; scrambled siRNA sense, 5′-CCU ACG CCA CCA AUU UCG U (dTdT)-3′; and scrambled siRNA antisense 5′-ACG AAA UUG UGG CGU AGG (dTdT)-3′. Approximately 3 × 10^4^cells were seeded in each well of the chamber slide glass and 2 × 10^5^cells were seeded in each well of a 6-well plate containing 10% FBS and were incubated overnight. Transfection was performed using Lipofectamine^®^ RNAiMAX (Invitrogen, Carlsbad, CA, United States) to obtain a final RNA concentration of 40 nM. After 48 h, the cells were treated with STK899704 for 24 h and were harvested for Western blot analysis or SA-β-Galactosidase staining.

### Statistical Analyses

Statistical analyses were performed using one-way analysis of variance (ANOVA) with Tukey’s honest significant difference (HSD) test. Three independent experiments were performed. The data are expressed as mean ± standard deviation (SD, *n* = 3).

## Results

### STK899704 Exhibits Anti-tumor Activity in Lung Cancer

Since STK899704 has been reported to have antitumor effect in a skin carcinogenesis model (Sakchaisri et al., 2017), we first used A549 lung cancer cells to generate the xenograft model by subcutaneous injection of A549 into mice. Mice which also received orally administered STK899704 showed decreased tumor weight (Supplementary Figure S1A). The tumors of the STK899704 group grew slower than vehicle treated control group (Supplementary Figure S1B). These data suggest that STK899704 has substantial antitumor activity in the *in vivo* A549 lung cancer cell xenograft model.

### STK899704 Induces Down-Regulation of Proliferation and Mitotic Catastrophe via Tubulin Aggregation in A549 Cells

**FIGURE 1:**
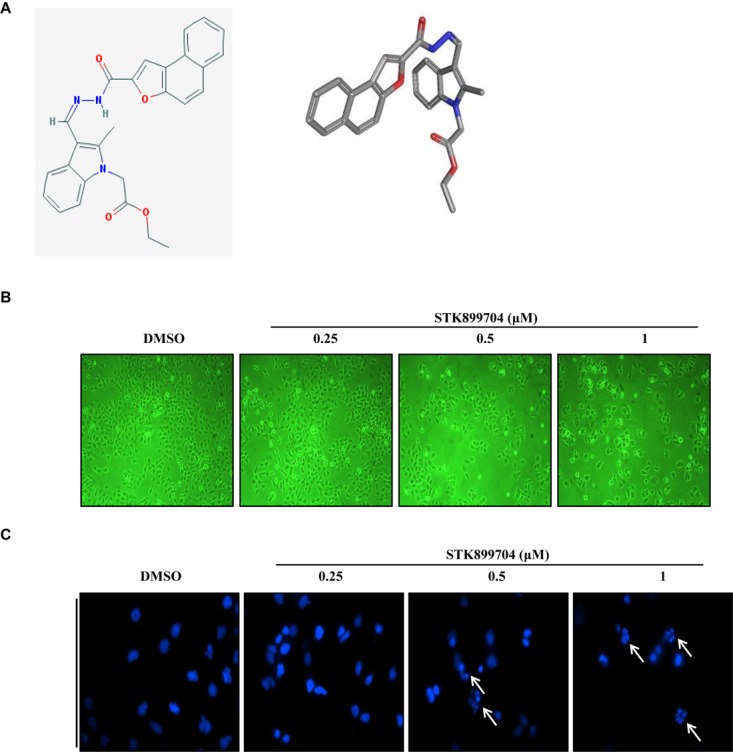
Cytotoxic effects of STK899704 on A549 cells. **(A)** Chemical structure of STK899704. **(B)** Micrographs of A549 cells treated with the indicated concentrations of STK899704. **(C)** STK899704-induced changes in nuclear morphology were determined by performing 4′,6-diamidino-2-phenylindole (DAPI) staining. Micrographs were taken using a phase-contrast microscope (magnification, 100×). The white arrows indicate cells showing mitotic catastrophe.

**FIGURE 2:**
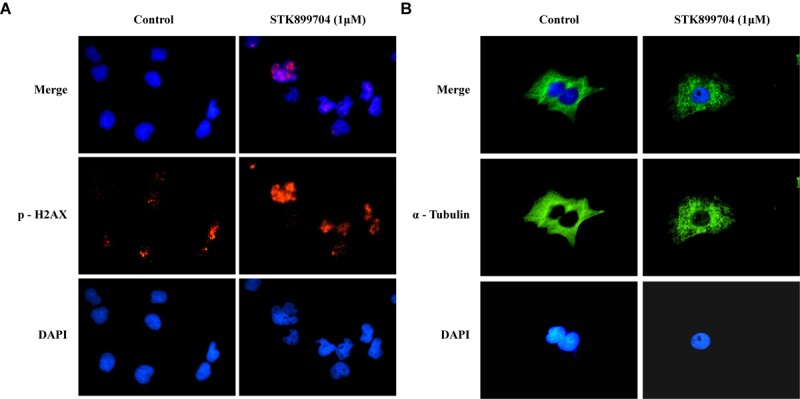
STK899704 induces α-tubulin aggregation and DNA damage. Immunofluorescence staining for a well-known DNA damage marker γ-H2A.X. α-tubulin was used as a control marker. A549 cells were treated with STK899704 for 24 h in a dose-dependent manner. **(A)** γ-H2A.X (phospho S139). **(B)** α-tubulin.

A previous study reported the antiproliferative effect of STK899704 on several cancer cell lines including lung cancer cells (Sakchaisri et al., 2017). STK899704 also displays anticancer activity against HeLa cervical cancer cells. In the present study, STK899704 induced mitotic arrest at 24 h and induced apoptotic cell death at 48 h. Based on these findings, we tried to reveal the anticancer mechanism of STK899704 (the chemical structure is shown in **Figure 1A**) on lung cancer cells. In the previous study, STK899704 inhibited A549 lung cancer cell growth with an IC_50_ of 0.84 μM at 96 h (Sakchaisri et al., 2017). Similar with this observation, STK899704 was cytotoxic to A549 cells with various concentrations and at various time points (Supplementary Figure S2). STK899704 showed similar cytotoxicity at different times, therefore, the short time point in subsequent experiments was 24 h. Also, STK899704 suppressed cell growth of other lung cancer cells, but not that of HaCaT normal human keratinocyte cells (Supplementary Figure S3). Consequently, we used A549 lung cancer cells to verify the effect and mechanism of STK899704. Optical microscopy analyses showed decreased proportion of viable STK899704 treated A549 cells (**Figure 1B**). As DNA damage is one feature for cell death, STK899704 treated A549 cells were subjected to DAPI staining, which showed altered nuclear morphology and detected mitotic catastrophe (**Figure 1C**). Mitotic catastrophe indicates cell death resulting from delayed mitosis. For more clear evidence, we conducted immunofluorescence staining for the well-known DNA damage markers γ-H2A.X and α-tubulin. The level of γ-H2A.X was increased in STK899704 treated cells (**Figure 2A**), as also occurred when cells suffered DNA damage (Kuo and Yang, 2008). A previous study suggested that tubulin is aggregated in HeLa cervical cancer cells (Sakchaisri et al., 2017). Tubulin aggregation can induce apoptosis, cell cycle arrest, DNA damage, and senescence (Gan et al., 2011; Demaria et al., 2017). In A549 cells, STK899704 also triggered α-tubulin aggregation (**Figure 2B**).

### STK899704 Inhibits Cell Cycle Arrest via G_2_/M Phase Arrest

**FIGURE 3:**
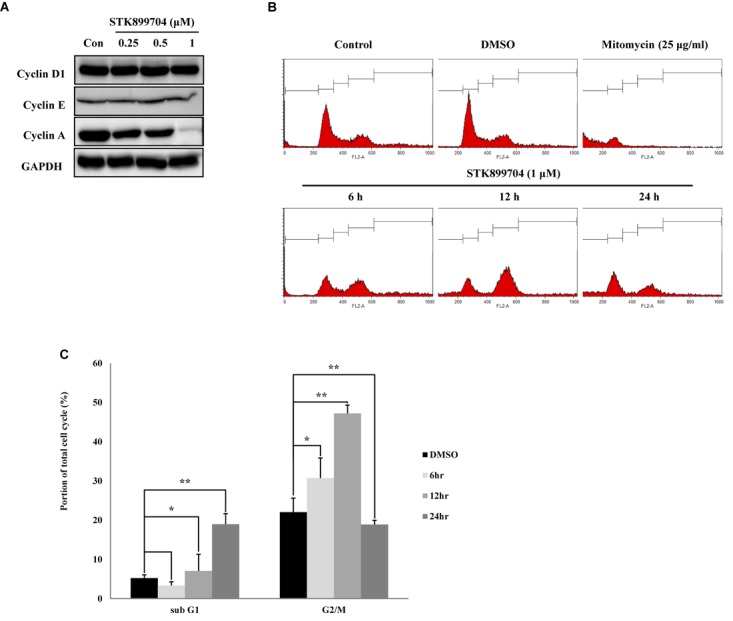
Effects of STK899704 on cell cycle regulation. Effects of STK899704 on cell cycle regulation. **(A)** Cells were treated with STK899704 for 24 h. Western blots of cyclins D, E, and A. GAPDH was used as an internal control. **(B)** Cells were treated with STK899704 for 0–24 h. Histogram of cell cycle distribution. STK899704 treated A549 cells were stained with PI and were counted using a flow cytometer (FACS analysis). **(C)** Bar graph for comparing the proportion of cells in the sub-G_1_ and G_2_/M phases. Data are presented as mean ± standard deviation (*n* = 3). ^∗^*P* < 0.05 and ^∗∗^*P* < 0.005 versus control cells.

STK899704 induces cell cycle arrest at the G_2_/M phase in HeLa cervical cancer cells (Sakchaisri et al., 2017). In the present study, whether the down-regulation of cell viability might be due to the mitotic arrest was investigated for the first time. To test this, the levels of the cyclin family of proteins were determined; these proteins are the core factors involved in cell cycle regulation. Western blot analysis showed a dramatic decrease in cyclin A, but not cyclin D1 and E levels in STK899704 treated cells (**Figure 3A**). Cyclin A regulates the S and G_2_/M phases of the cell cycle along with the cofactors CDK1 and CDK2, respectively. PI staining was conducted to analyze cell cycle arrest. Similar with previous findings, STK899704 up to 0.5 μM increased the number of cells in G_2_/M phase, but the proportion of cells in the G_1_ and S phases were decreased, whereas 1 μM STK899704 increased the sub-G_1_ population (Supplementary Figure S4). As the proportion of cells in the G_2_/M phase increased slightly, the PI staining procedure was repeated with time. Comparative analyses of each treatment revealed increasing G_2_/M phase arrest until 12 h and increasing sub-G_1_ arrest at 24 h (**Figures 3B,C**). For a comparative study of apoptosis, cells were treated with mitomycin C as an apoptosis inducer. Mitomycin C and STK899704 treated cells showed different patterns at 24 h. Sub-G_1_ was increased with both treatments, but G_2_/M phase arrest was only identified in cells treated with STK899704 (**Figure 3B**). These results confirm that STK899704 induces G_2_/M phase arrest by inhibiting cyclin A expression. Taken together, these findings suggest that STK899704 might cause cell cycle arrest of A549 cells by inducing DNA damage responses via tubulin aggregation.

### STK899704 Induces Cell Death Independent of Intrinsic and Extrinsic Apoptotic Pathways

**FIGURE 4:**
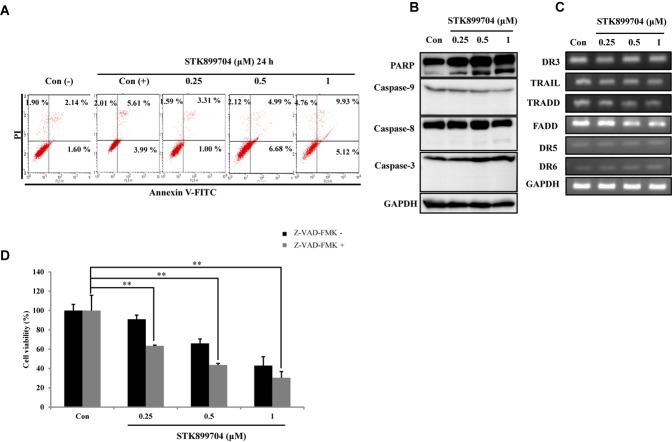
Effect of STK899704 on apoptosis in A549 cells. Effect of STK899704 on apoptosis in A549 cells. A549 cells were treated with STK899704 for 24 h in a dose-dependent manner. **(A)** Apoptotic cells were determined by performing annexin V-PI staining by FACS. Quadrant 1 contains late apoptotic cells (annexin V and PI positive), and quadrant 4 contains early apoptotic cells (annexin V positive and PI negative). **(B)** Western blotting was performed to measure the levels of apoptosis-related proteins. GAPDH was used as an internal control. **(C)** The mRNA levels of genes encoding death receptors and their ligands were analyzed by performing Reverse transcription polymerase chain reaction (RT-PCR). *GAPDH* was used as an internal control. **(D)** A549 cells were treated with STK899704 with Z-VAD-FMK (total caspase inhibitor) for 24 h. Cell viability was determined by performing CCK-8 assay. Data are presented as mean ± standard deviation (*n* = 3). ^∗∗^*P* < 0.005 versus control cells.

We attempted to clarify whether the STK899704-mediated cell death was due to apoptosis. In the previous study, STK899704 could induce apoptosis in HeLa cells upon 48 h incubation, but not at 24 h. Presently, annexin V–PI staining assay with A549 cells revealed non-significant alterations in the levels of early and late apoptotic markers at both 24 and 48 h (**Figure 4A**). To determine whether the down-regulation of proliferation was dependent on caspase, we checked caspase and PARP protein expression using western blot analysis. Elevated PARP cleavage was observed with STK899704 treatment, which is associated with DNA damage repair and aging (Piskunova et al., 2008). However, levels of caspase family proteins (the primary factors of apoptotic cell death) were unaltered in STK899704 treated cells (**Figure 4B**). Likewise, the death receptor family proteins, which are associated with the extrinsic apoptotic pathway, were unaltered in STK899704 treated cells (**Figure 4C**). We treated pan (total) caspase inhibitor before STK899704 treatment and performed the cell viability assay. As shown in **Figure 4D**, the pan (total) caspase inhibition assay did not show a recovery of cell viability. Collectively, these results indicate that STK899704 induced the down-regulation of A549 cell proliferation independent of the caspase-mediated apoptotic pathways.

### STK899704 Induces Cellular Senescence Through the p53 Pathway

**FIGURE 5:**
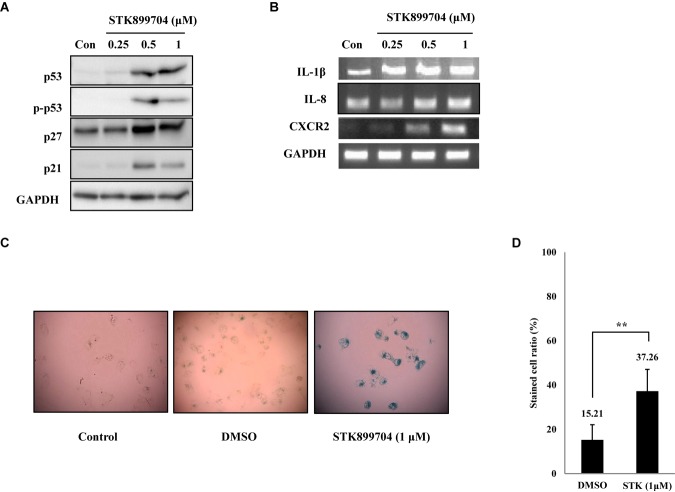
STK899704 induces senescence in A549 lung cancer cell line. **(A)** Western blots of p53, phosphorylated p53, p27, and p21. **(B)** The mRNA levels of genes encoding SASP-associated proteins were analyzed by performing RT-PCR. **(C)** β-Galactosidase staining. **(D)** Bar graph for measuring the proportion of β-galactosidase-stained cells. Data are presented as mean ± standard deviation (*n* = 3). ^∗^*P* < 0.05 and ^∗∗^*P* < 0.005 versus control cells.

**FIGURE 6:**
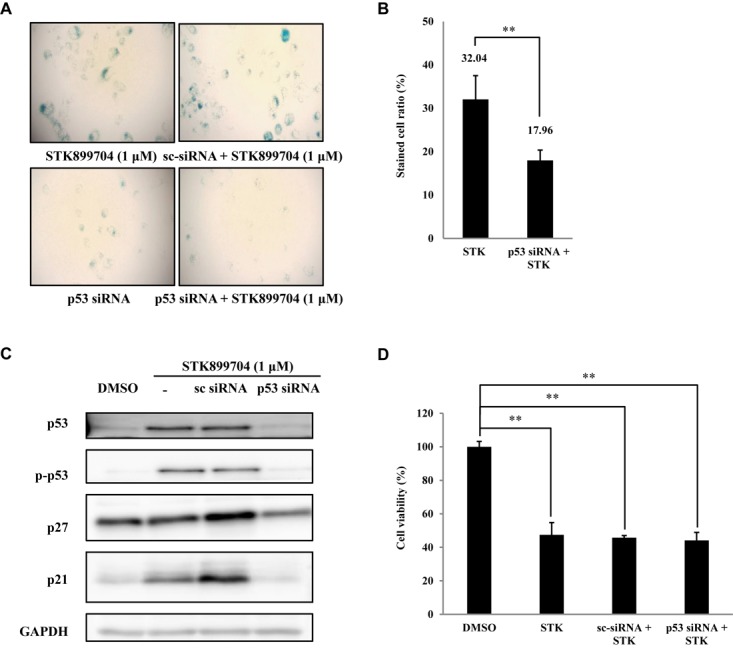
Effects of STK899704 on p53-mediated senescence. **(A)** Inhibition of p53 expression by using *p53* siRNA. Representative image of STK899704 treated β-galactosidase-stained A549 cells. **(B)** Bar graph for measuring the proportion of β-galactosidase-stained cells. **(C)** Western blots of p53, phosphorylated p53, p27, and p21. GAPDH was used as internal controls. **(D)** Cell viability assay using *p53* siRNA. Data are presented as mean ± standard deviation (*n* = 3). ^∗^*P* < 0.05 and ^∗∗^*P* < 0.005 versus control cells.

**FIGURE 7:**
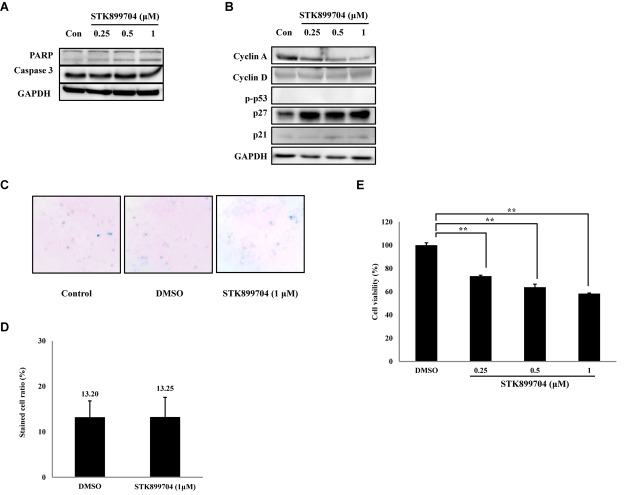
Effects of STK899704 on apoptotic related factors, cyclins and β-galactosidase staining in NCI-H1299 cells. **(A)** Effect of STK899704 on apoptosis in NCI-H1299 cells. NCI-H1299 cells were treated with STK899704 for 24 h in a dose-dependent manner. Western blotting was performed to measure the levels of apoptosis-related proteins. GAPDH was used as an internal control. **(B)** Cells were treated with STK899704 for 24 h. Western blots of cyclins D, E, and A. GAPDH was used as an internal control. **(C)** β-Galactosidase staining. **(D)** Bar graph for measuring the proportion of β-galactosidase-stained cells. Data are presented as mean ± standard deviation (*n* = 3). ^∗^*P* < 0.05 and ^∗∗^*P* < 0.005 versus control cells. **(E)** Effect of STK899704 on the viability of NCI-H1299 cells. The cells were incubated with various concentrations of STK899704 (0.25–1 μM) for 24 h.

**FIGURE 8:**
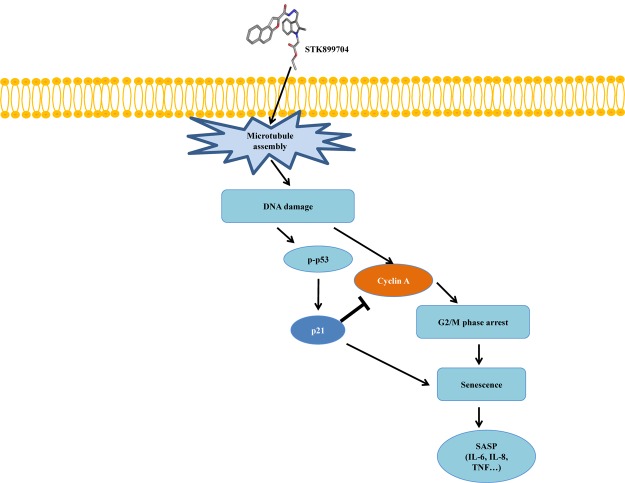
Mimetic diagram of STK899704 efficacy pathway in A549 cells. STK899704 induces cell cycle arrest and senescence through a p53-associated pathway. STK899704 can induce tubulin aggregation, suffering DNA damage and p53 is activated. Mitosis arrest is induced by p53 and followed by cellular senescence.

As p53 is an essential factor regulating the DNA damage response, we hypothesized that STK899704-induced cellular senescence is mediated via the p53 pathway. As shown in **Figure 5A**, treating A549 cells with STK899704 resulted in elevated levels of p53 and phosphorylated p53. In addition, STK899704 treatment enhanced the levels of p27 and p21 (**Figure 5A**). p53 promotes cell death by inducing apoptosis, autophagy, ROS response, and senescence (Levine and Oren, 2009). To analyze cell death, these possibilities were examined. Autophagy and ROS stress signals did not match with the observations (Supplementary Figures S5, S6). PCR was conducted to analyze the expression of genes encoding SASP-associated proteins. Senescent cells display SASP, which is characterized by the production of IL-1β, IL-8, CXCR2, and other molecules. They are characteristic of induced senescence. As anticipated, SASP-associated factors were upregulated by STK899704 in A549 cells (**Figure 5B**). β-Galactosidase staining was also performed to determine cellular senescence. An increased proportion of β-galactosidase-staining was observed in STK899704 treated A549 cells (**Figure 5C**), with the proportion of stained cells being more than double of that of control cells (**Figure 5D**). For more clear evidence between p53 and senescence, p53 expression was inhibited in A549 cells using *p53* siRNA. As expected, the silencing of p53 led to the decrease in the ratio of β-galactosidase-stained STK899704 treated A549 cells compared with that of control cells (**Figures 6A,B**). Western blot analysis showed a decrease in p53 and phosphorylated p53 levels in *p53* siRNA-transfected cells. The level of p21, but not p27, was also decreased (**Figure 6C**). However, the STK899704 mediated cell viability decrease was not recovered by p53 siRNA (**Figure 6D**). To explain this unexpected result, we used the NCI-H1299 p53 null lung cancer cell line. Consistent with the above finding, STK899704 did not induce caspase-3 cleavage in these cells, but induced PARP cleavage, expression of p21 and p27 expression, and inhibited cyclin A (**Figures 7A,B**). In the absence of p53, STK899704 could not induce cellular senescence of NCI-H1299 cells (**Figures 7C,D**), but did inhibit cell viability (**Figure 7E**). Thus, these data indicate that STK899704 induces cellular senescence via the p53 pathway, and subsequently induces partial cell death. The collective data indicate that STK899704 induces DNA damage, cyclin A related cell cycle arrest, and p53-related senescence, leading to cell death. A model summarizing the effects of STK899704 is presented in **Figure 8**.

## Discussion

Despite the many efforts in anticancer development, novel anticancer agents remain urgently required. A previous study reported the activity of STK899704 as a tubulin inhibitor possessing acyl hydrazone moiety, which is a useful scaffold for anticancer drug development. STK899704 showed antimitotic and apoptotic activities on HeLa cervical cancer cells (Sakchaisri et al., 2017). But, the effect and precise mechanism of STK899704 on lung cancer remained unclear. The present study reports for the first time that the microtubule destabilizing agent STK899704 induced DNA damage and inhibited the proliferation of A549 NSCLC cells. Unlike a previous finding, STK899704 showed antitumor activity by inhibiting proliferation, rather than inducing apoptosis, of A549 lung cancer cells. STK899704 induced mitotic catastrophe which involves the abnormal regulation of mitosis and is a feature of cell death and senescence (Gu et al., 2017) via tubulin aggregation (**Figure 2B**). These results suggest that STK899704 could induce mitotic arrest of A549 cells at 24 h. Therefore, we focused on cell cycle arrest and mitotic catastrophe as the reasons for STK899704 induced cell death. Western blotting and PI staining performed showed that STK899704 treatment decreased cyclin A, which regulates the S and G_2_/M phases of the cell cycle along with CDK1 and CDK2, respectively (**Figure 3A**). STK899704 treatment lead to the accumulation of cyclin B1 and Plk1 in HeLa cells (Sakchaisri et al., 2017), whereas it decreased cyclin A in A549 cells (**Figure 3A**). These results suggest that STK899704 promotes the decrease of cyclin A level, leading to the induction of abnormal mitosis, which in turn promotes mitotic catastrophe, cell death, and senescence. Along with this observation, the proportion of STK899704 treated A549 cells in the G_2_/M phase increased consistently until 12 h, while that in the sub-G_1_ phase increased up to 24 h. It can be suggested that prolonged mitotic arrest, leading to time-dependent increase of sub-G_1_ phase. Thus, we explored the apoptotic effect of STK899704 in lung cancer cells. STK899704 did not induce apoptosis upon 24 h incubation in A549 (p53 wild type) and NCI-H1299 (p53 null cells) NSCLC cells (**Figures 4, 7**). Detection of cleaved PARP suggested that DNA damage might be a reason for the STK899704 induced down-regulation of proliferation. To explain this phenomenon, we explored cellular senescence because cell cycle arrest in the sub-G_1_ phase is a well-known signal of cell death (Gong et al., 1994), and senescence is induced at G2/M phase arrest (Serrano et al., 1997). Based on the evidence from these result, it was elucidated the relationship of STK899704 and mitotic related factors. STK899704 treatment increased p53, phosphorylated p53, p21, and p27 levels (**Figure 5A**), which may be due to the decrease in cyclin A level and G_2_/M phase arrest because of tubulin aggregation. These data supported already reported results regarding on the causes of senescence (Roninson et al., 2001; Tierno et al., 2009; Chou et al., 2016). Also, β-Galactosidase staining showed that STK899704 induced senescence in A549 cells compared with that in control cells. To verify p53 mediated cellular senescence by STK899704, it was examined the relationship between STK899704 and p53 by inhibiting p53 expression with *p53* siRNA. First time, senescence seemed to be a key point of STK899704 induced cell death because it is one of the reasons of cell death (Chiantore et al., 2009). However, siRNA-induced inhibition of p53 did not alter cell viability, as determined by CCK-8 assay, meaning that STK899704 could trigger p53 mediated senescence which can be partially a cause of cell death. Based on this observation, STK899704 inhibits microtubule assembly, subsequently induce DNA damage and then, induces p53 mediated senescence.

## Conclusion

These data proved that the novel small molecule STK899704 is a potential agent for treating human lung cancers, causing cell cycle arrest, senescence, and cell death via tubulin aggregation. Thus, this report suggests that STK899704 can be a putative anti-cancer agent on lung cancer.

## Author Contributions

D-YY has generated the idea for the experiment and has corrected the manuscript. C-WP and YB have conducted the experimental work and written the manuscript. GS, JH, NS, and BK were in charge of chemical synthesis. J-HY and JTH have helped during the experimental work and in the interpretation of the results. All authors read and approved the final manuscript.

## Conflict of Interest Statement

The authors declare that the research was conducted in the absence of any commercial or financial relationships that could be construed as a potential conflict of interest.
