# Supplementary material for: The Novel Small Molecule STK899704 Promotes Senescence of the Human A549 NSCLC Cells by Inducing DNA Damage Responses and Cell Cycle Arrest
Source: Front Pharmacol. 2018 Apr 16;9:163. doi: 10.3389/fphar.2018.00163 (PMC5912185; doi:10.3389/fphar.2018.00163)
Supplement: Supplementary file 1 [file Data_Sheet_1.pdf]

Supplementary Figure 1

A

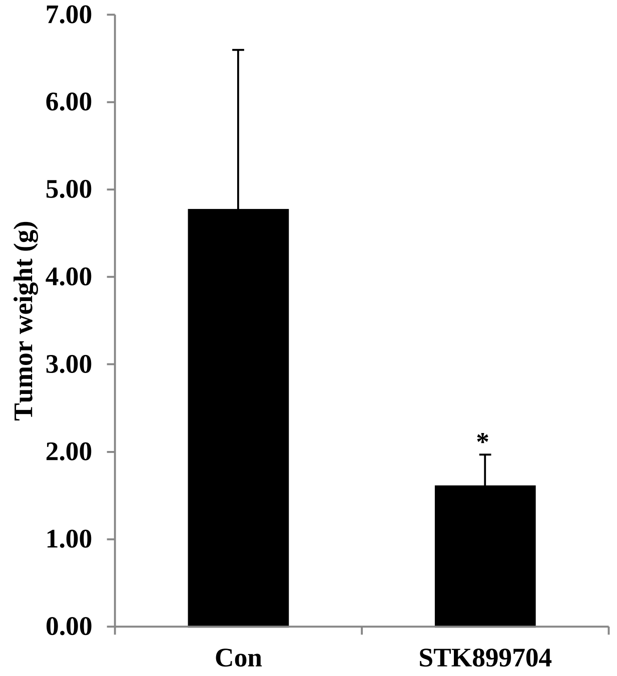

B

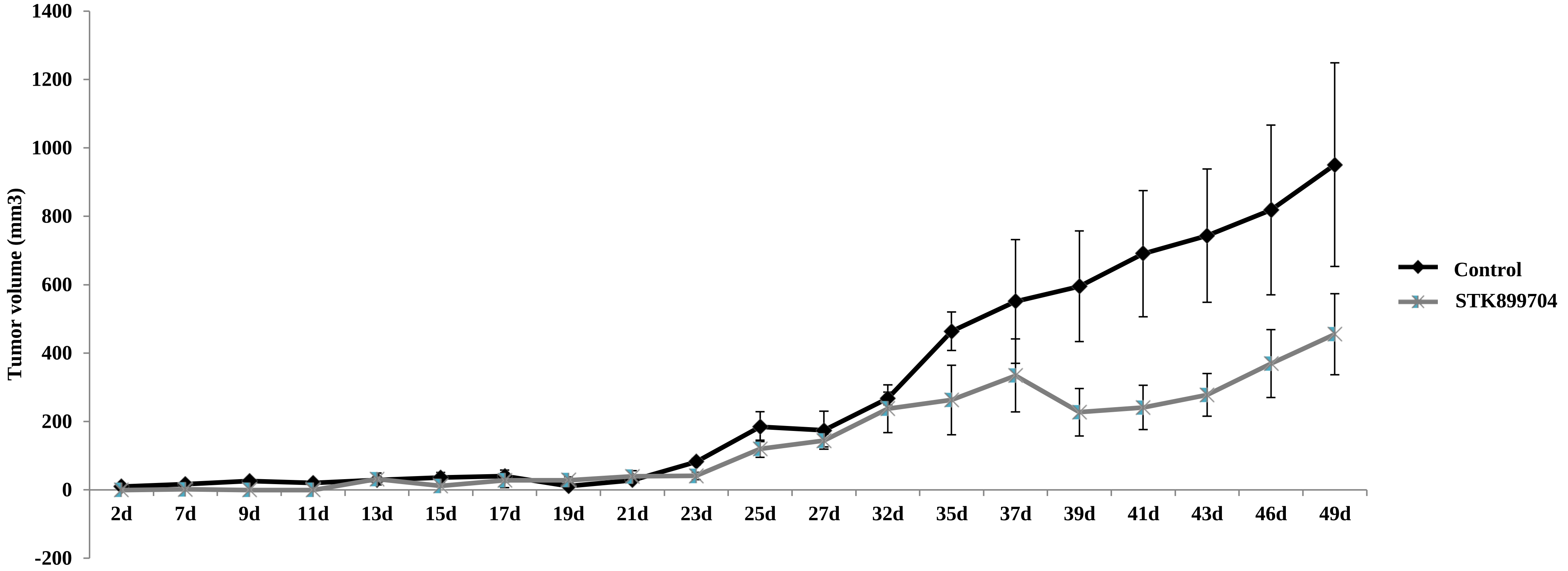

Figure S1. Effects of STK899704 on tumor growth. Male BALB/c nude mice (5 weeks old) were acclimated for 1 week and the mice (each group n= 4) were inoculated subcutaneously in the back with a suspension (5 million cells/100 µl) of A549 cells. (A) Tumor volume was calculated by tumor volume = (a × b square)/2, where a = length, b = width. The a and b values were measured twice a week using digital Vernier calipers. (B) Tumor tissues were weighed immediately after mice sacrifice. *P < 0.05 versus control mice.

Supplementary Figure 2

A

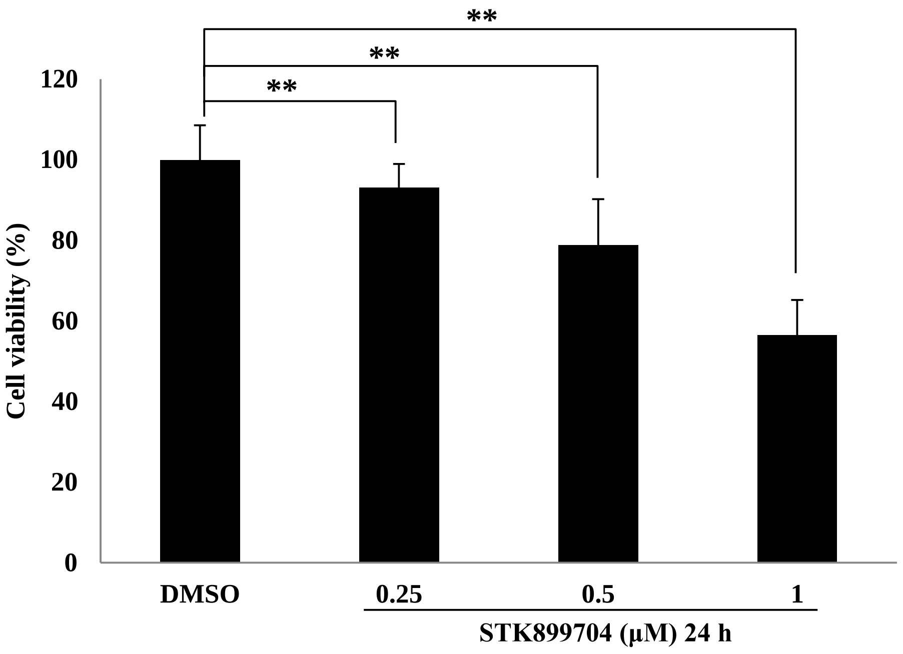

B

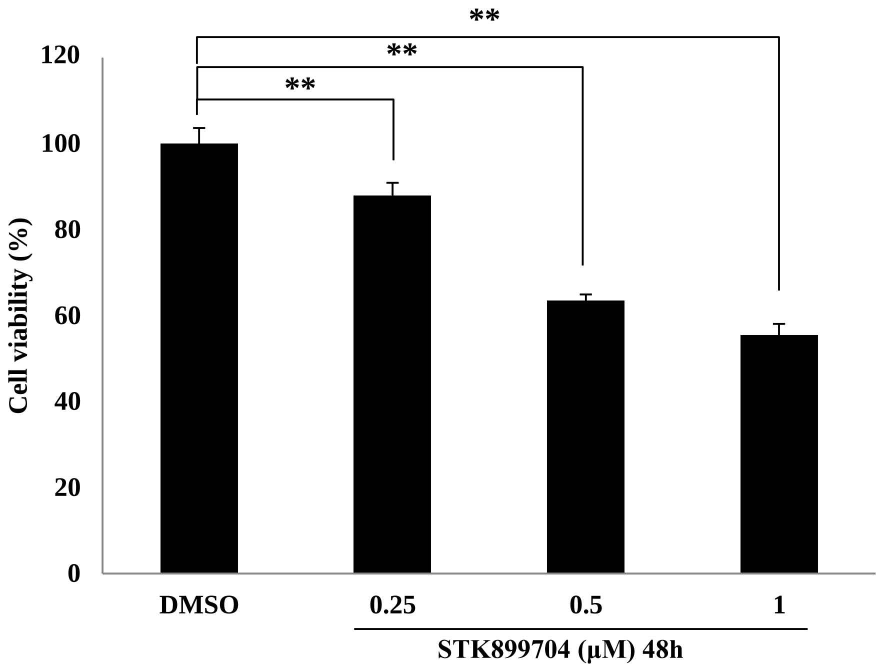

C

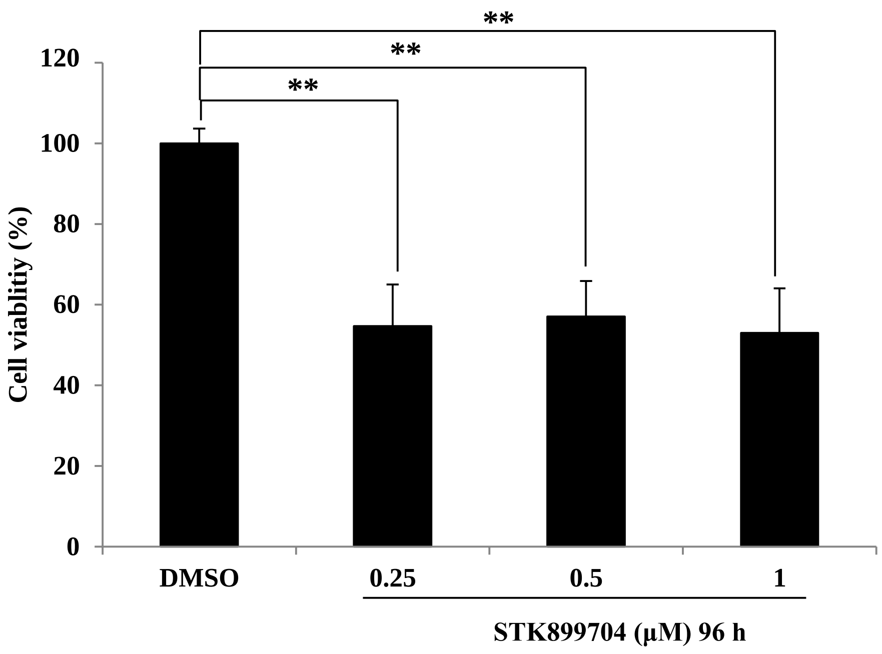

Figure S2. Effect of STK899704 on the viability of A549 cells. A549 cells were incubated with various concentrations of STK899704 (0.25–1 µM) for 24, 48, 96 h.

Supplementary Figure 3

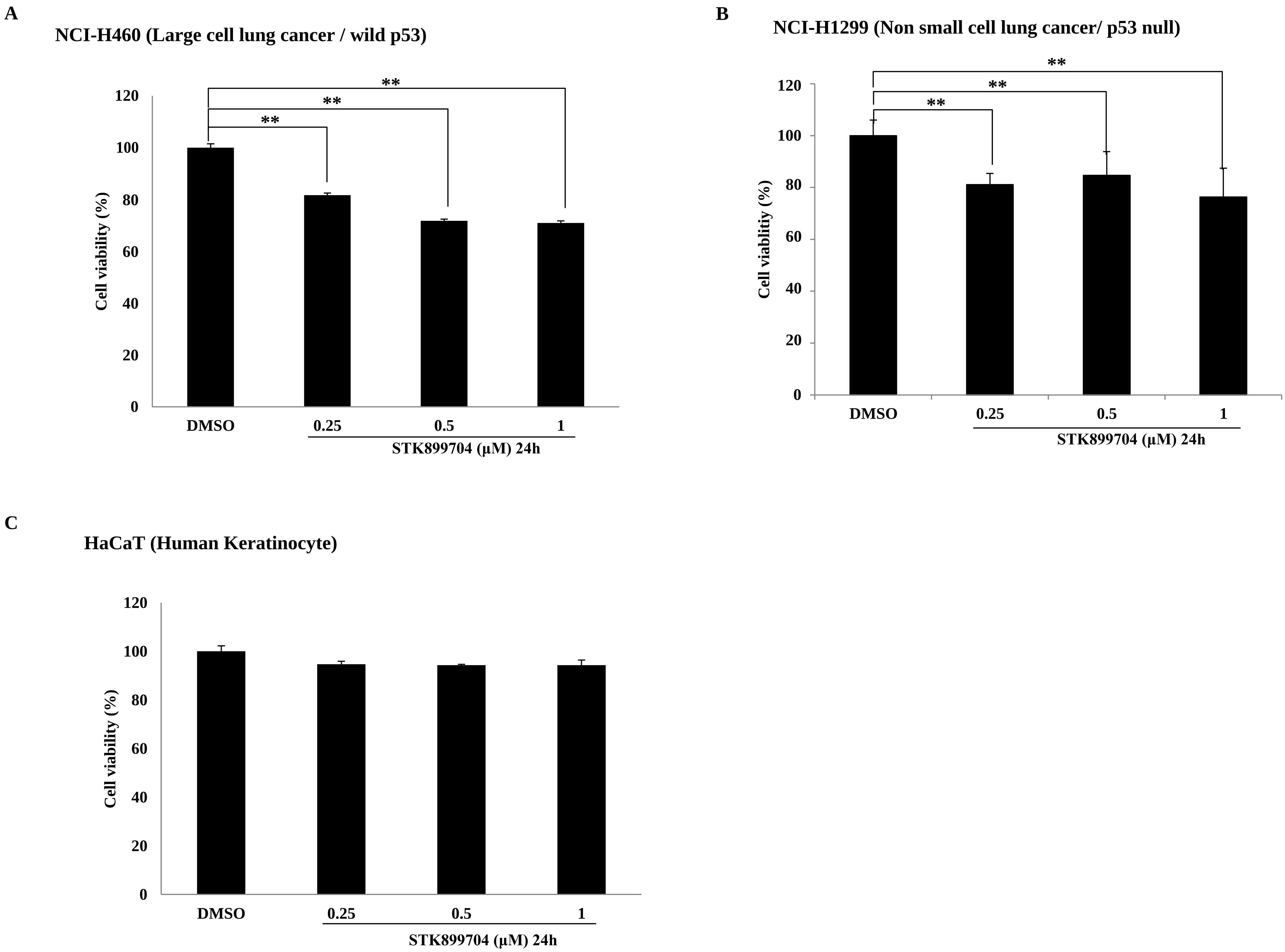

Figure S3. Effect of STK899704 on the viability of several cell lines. The cells were incubated with various concentrations of STK899704 (0.25–1 μM) for 24 h.

Supplementary Figure 4

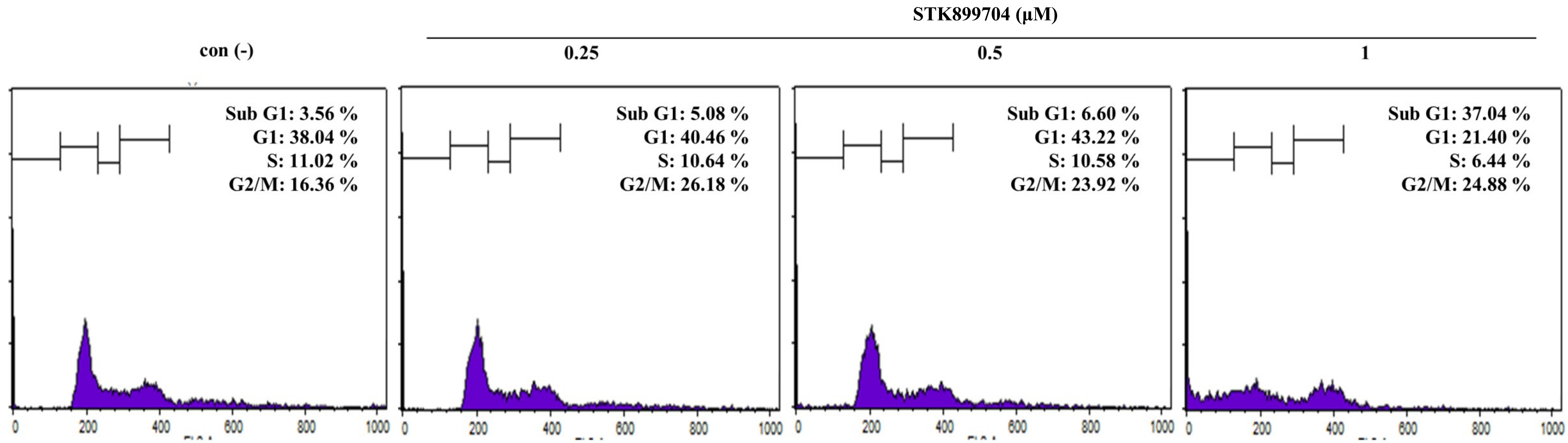

Figure S4. Cell cycle analysis was conducted by performing PI staining by FACS. A549 cells were treated with STK899704 for 24 h. Cells treated with 1 μM STK899704 were arrested in the sub-G1 and G2/M phases compared with control cells.

Supplementary Figure 5

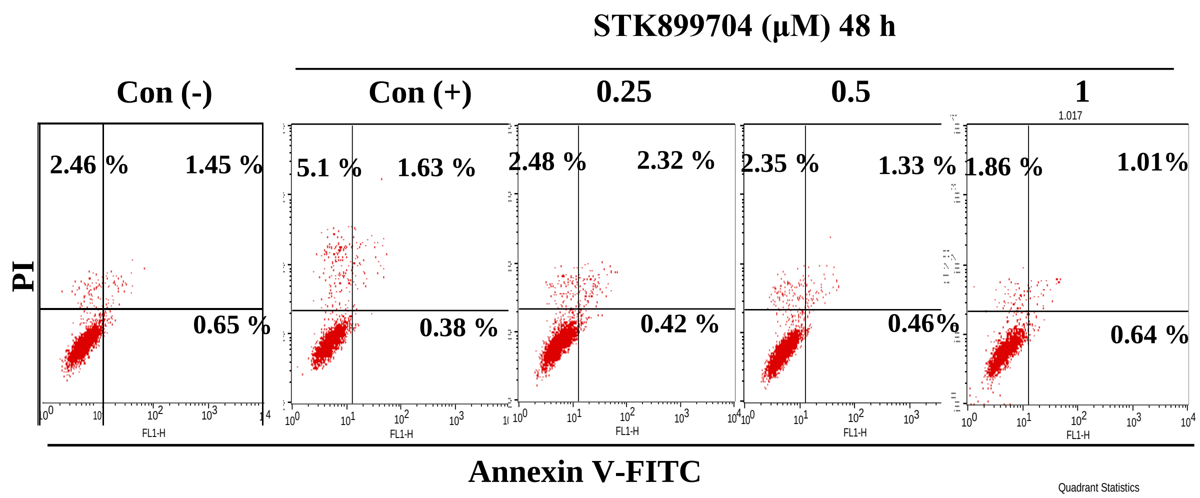

Quadrant Statistics

File: 1.017 | Log Data Units: Linear Values
Sample ID: | Patient ID:
Tube: Untitled | Panel: Untitled Acquisition Tube Lis
Acquisition Date: 21-Dec-17 | Gate: G1
Gated Events: 2957 | Total Events: 10000
X Parameter: FL1-H (Log) | Y Parameter: FL2-H (Log)
Quad Location: 13, 21

Quad	Events	% Gated	% Tota	X Mean	X Geo Mean	Y Mean	Y Geo Mean
UL	55	1.86	0.55	7.26	6.59	36.02	33.98
UR	30	1.01	0.30	26.97	24.91	45.24	43.34
LL	2853	96.48	28.53	5.06	4.63	5.96	5.41
LR	19	0.64	0.19	15.93	15.75	12.92	12.55

Figure S5. Effect of STK899704 on apoptosis in A549 cells for 48 h. A549 cells were treated with STK899704 for 48 h in a dose-dependent manner. (A) Apoptotic cells were determined by performing annexin V-PI staining by FACS. Quadrant 1 contains late apoptotic cells (annexin V and PI positive), and quadrant 4 contains early apoptotic cells (annexin V positive and PI negative).

Supplementary Figure 6

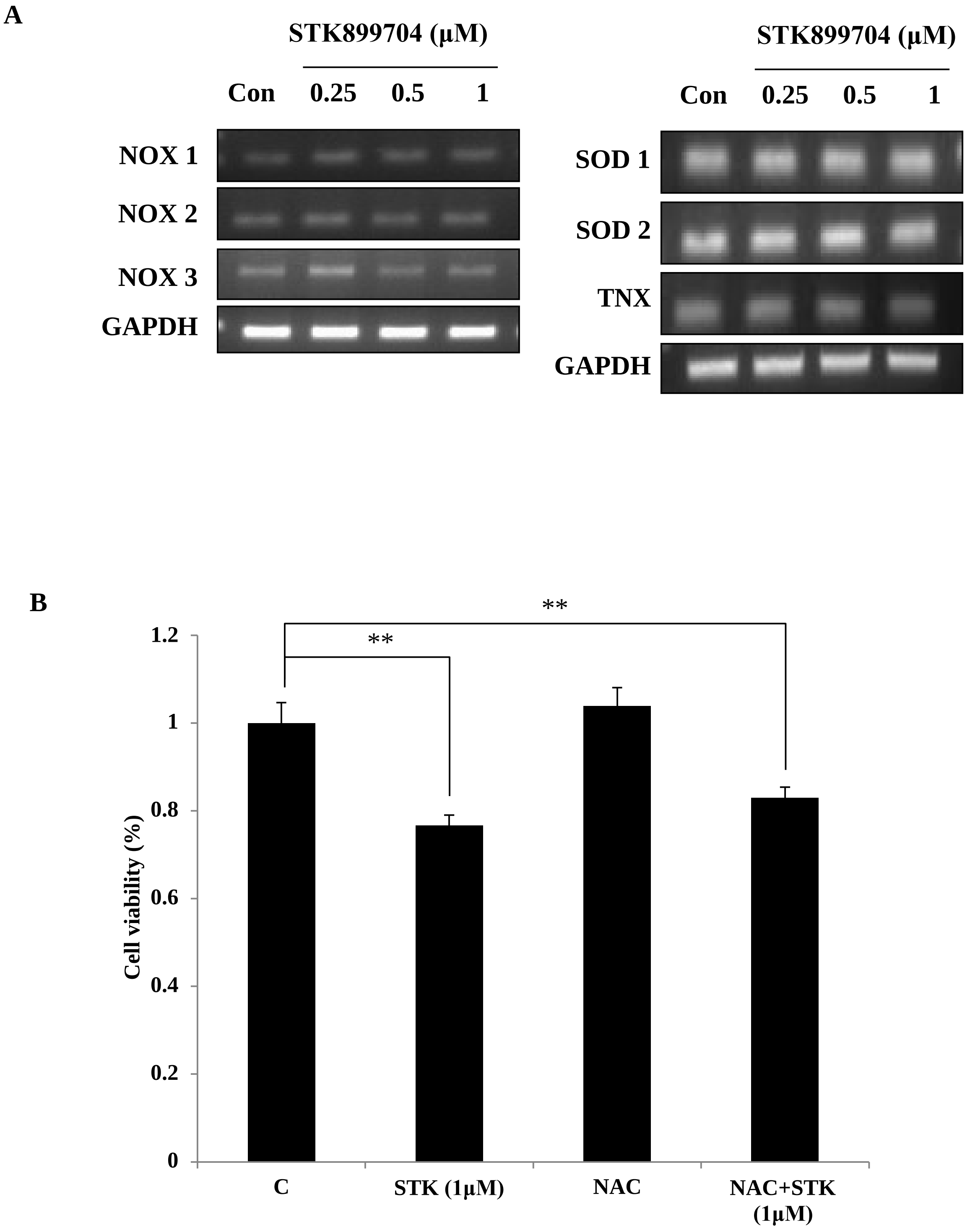

Figure S6. Analysis of ROS signalling-related factors. **(A)** ROS signalling-related factors were determined by performing PCR. Primer sets used were the same as tho se used in a previous study 1. Full length gels are located beside. **(B)** The viability of A549 cells treated with STK899704 and an ROS inhibitor (NAC). Data are prese nted as mean ± standard deviation (n = 3). *p < 0.05 and **p < 0.005 versus control cells.

Supplementary Figure 7

A

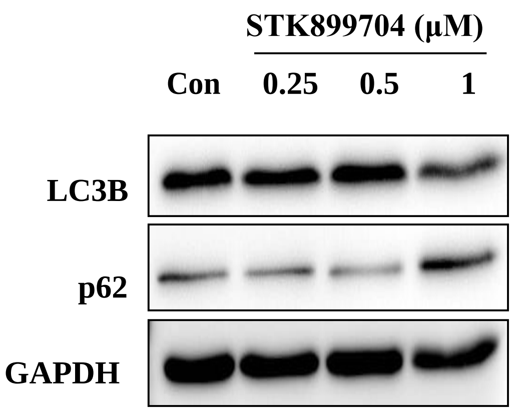

B

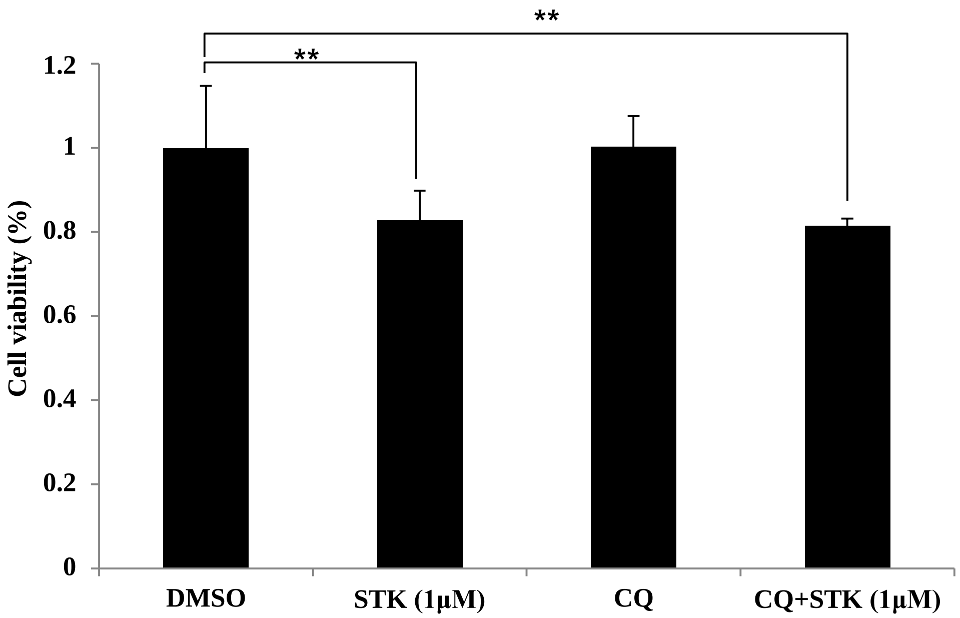

Figure S7. Analysis of autophagy-related factors. **(A)** Autophagy-related factors were determined by western blotting. Full length blots were located beside. **(B)** The viability of A549 cells treated with STK899704 and an autophagy inhibitor (chloroquine diphosphate salt; CQ). Data are presented as mean ± standard deviation (n = 3). *p < 0.05 and **p < 0.005 versus control cells.
